# Eradication of enterococci biofilms by lactic acid alone and combined with chlorhexidine and cetrimide

**DOI:** 10.4317/medoral.18133

**Published:** 2012-05-01

**Authors:** María T. Arias-Moliz, Pilar Baca, Santiago Ordóñez-Becerra, María P. González-Rodríguez, Carmen M. Ferrer-Luque

**Affiliations:** 1 DDS, PhD: Assistant Professor. Department of Microbiology, School of Dentistry. University of Granada, Campus de Cartuja, Colegio Máximo s/n, Granada, Spain; 2 DDS, MD, PhD: Professor. Department of Preventive Dentistry, School of Dentistry. University of Granada, Campus de Cartuja, Colegio Máximo s/n, Granada, Spain; 3 BDS: Postgraduate Student, DDS, PhD: Associate Professor, DDS, MD, PhD: Associate Professor. Department of Operative Dentistry, School of Dentistry. University of Granada, Campus de Cartuja, Colegio Máximo s/n, Granada, Spain

## Abstract

Objective: The antimicrobial activity of lactic acid (LA) alone or in combination with chlorhexidine (CHX) and cetrimide (CTR) against three Enterococcus faecalis strains, E. faecalis ATCC 29212, E. faecalis EF-D1 and E. faecalis U-1765, one Enterococcus durans strain and one dual-species biofilm was investigated. Study Design: The irrigating solutions tested were 20%, 15%, 10%, 5% and 2.5% LA, alone and in combination with 2% CHX and with 0.2% CTR. The biofilms were grown in the MBECTM high-throughput device for 24 hours and exposed to the solutions for 30 seconds and 1 minute. “Eradication” was defined as 100% bacterial kill. Results: Twenty percent LA eradicated all enterococci biofilms after 30 seconds contact time. The association of LA + 0.2% CTR achieved better results than LA alone, in contrast with the results obtained using LA + 2% CHX. E. durans was eradicated by all the tested solutions at 1 minute. The dual-species biofilm, E. faecalis ATCC 29212 + E. durans, gave intermediate values of the pure cultures. Conclusions: LA is capable of eradicating enterococci biofilm at a concentration of 20%. The combination of lower concentrations with 0.2% CTR achieved eradication after 1 minute.

** Key words:**Biofilm, cetrimide, chlorhexidine, enterococcus durans, enterococcus faecalis.

## Introduction

The success of root canal treatment depends on the control of microorganisms present in infected root canals (1). Enterococci are commonly isolated bacteria in root canal systems with failed endodontic treatment due to their ability to adhere to dentin and invade dentinal tubules and to form communities organized in biofilms, which may contribute to bacterial resistance and persistence after intracanal anti microbial procedures (2). The complexity of the root canal system makes complete elimination of bacteria by means of chemo-mechanical treatment nearly impossible (3).

No single irrigant is capable of exerting anti microbial activity, removing the smear layer, and dissolving organic debris. For this reason, different agents are used in combined form in order to achieve such properties (4). The most widely used anti microbial agents are sodium hypochlorite and chlorhexidine (CHX), the former outstanding for its ability to dissolve necrotic tissue, and the latter for its substantivity (5). Chelating agents -such as EDTA, citric and maleic acids-have been used in view of their efficacy in eliminating the smear layer (6,7). Maleic acid itself has the capacity to eradicate Enterococcus faecalis biofilms when applied at a clinical concentration of 7%. However, the anti microbial activity of EDTA and citric acid against biofilms is controversial (8). The combination of the two acids with surfactant agents such as cetrimide (CTR) has nonetheless demonstrated a greater anti microbial efficacy (9).

The use of lactic acid (LA) as an irrigating solution was introduced by Ayad et al. (10), who showed that it can remove the smear layer, improve the bond strength of the obturation material in contact with the root canal walls (11,12) while also increasing the fracture resis-tance of thin-walled endodontically treated teeth (13). It is an organic acid that occurs naturally in the muscles during muscular exercise, suggesting it is more biologically acceptable than other chelating agents, and its price makes it economically competitive with endodontic irrigants (11). Due to its nature as an organic acid, it exerts ant microbial activity against food borne bacteria (14). However, its action against enterococci strains is unknown, as is its effect when combined with ant imicrobial and surfactant agents. The aim of the present study was therefore to evaluate, in vitro, the anti microbial activity of LA alone or in combination with CHX and CTR against three E. faecalis strains, one E. durans strain and one dual-species biofilm.

## Material and Methods

Microorganisms and tested materials

The enterococci strains used in this study and their source were: E. faecalis ATCC 29212, E. faecalis EF-D1 and E. durans ED-C1 from the collection of the Microbiology Laboratory, School of Dentistry, University of Granada, obtained from failed endodontic treatment; and E. faecalis U-1765 from a human nosocomial infection, provided by the Microbiology Laboratory, School of Science, University of Granada. Bacterial strains were taken from a 4ºC stock culture and streaked out twice on BHI (Brain Heart Infusion; Scharlau Chemie S.A., Barcelona, Spain) agar plates for 24 hours at 37ºC. Colonies were suspended in BHI to obtain a 1 McFarland initial bacterial suspension of approximately 3×108 colony-forming units per mL (CFU/mL). All strain cultures were checked for purity by Gram stain and colony morphology.

The test agents and concentrations assayed were: 20%, 15%, 10%, 5% and 2.5% LA (Panreac, Castellar del Valles, Spain), alone and in combination with 2% CHX (Guinama, Alboraya, Valencia, Spain) and with 0.2% CTR (Sigma-Aldrich Chemie, Steinheim, Germany). All the dilutions were carried out using sterile distilled water and were stored at room temperature until use, for no more than 60 minutes.

Biofilm susceptibility test

The biofilm model used in this study was the MBECTM-high-throughput (HTP) device (Innovotech, Edmonton, Alberta, Canada) (8,15). To summarize briefly, there are two parts to this batch-culture apparatus. The top half is a lid with 96 pegs that also fits over a standard 96-well micro titer plate. The bottom half is a fluted trough that guides inoculated growth medium across the pegs when the device is placed on a rocker (16). The initial bacterial suspension was diluted 30-fold in BHI broth (Scharlau Chemie S.A., Barcelona, Spain) (approximately 107 CFU/mL), and 22 ml of the 1 in 30 dilution was used to inoculate the trough of the MBECTM-HTP device. Simple biofilms of each of the enterococci strains were created, and one dual-species biofilm was formed through the association of E. faecalis ATCC 29212 and E. durans ED-C1. The peg lid was fitted inside the trough and the assembled device was then placed on a rocking table (OVAN, model Swing Sw 8 10000-00015, Badalona, Spain) and incubated at 37°C for 24 hours, at 5 rocks per minute. The shear force of the rocking motion facilitated the formation of 96 equivalent biofilms on the pegs. Biofilms forming on the lid of the MBECTM-HTP device were rinsed by inserting the peg into a micro titer plate with 0.9% saline solution per well for 2 minutes. To determine biofilm formation, four pegs were broken off from each device, placed in 200 µl 0.9% saline, and sonicated on a water-table sonicator (Branson, model 5510E-MT, Danbury, CT, USA) for 10 minutes (8). The disrupted biofilms were diluted serially and plated for viable cell counting. This growth control was used to evaluate the initial number of bacteria formed in the biofilm in each assay.

The susceptibility tests were done in a microtiter plate known as the ‘challenge plate’ (Nunclon Delta Surface, Nunc, Roskilde, Denmark). The volume of the wells of the challenge plate must be sufficient to submerge the peg past the height of the biofilm produced. We used flat-bottom 96-well micro titer plates (Nunclon Delta Surface; Nunc, Roskilde, Denmark) with a final volume of 200 μl. The irrigating solutions were placed along the length of the challenge plate, allowing the first and the last wells of each row, containing BHI, to respectively serve as the sterility or negative, and growth or positive controls. The peg lid was submerged in the challenge plate for 30 seconds and 1 minute. Following exposure, the biofilms were rinsed twice as previously described to remove loosely adherent planktonic bacteria, and they were then placed in a micro titer recovery plate with 200 μl of BHI per well and sonicated for 10 mi-nutes. Disrupted biofilm cultures were diluted serially and plated for viable cell counting. Five replicates per irrigant concentration, bacteria biofilm and exposure time were performed in 3 different peg lids.

The effect of each test agent on the biofilm was determined by calculating the percentage of viable bacteria kill respect to the control as follows: [1-(mean CFUirrigant/mean CFUinitial bacterial number)] x 100. The term ‘eradication’ was used to denote the death of 100% of the bacterial population.

## Results

The results of the antibacterial effects of varied dilution`s of LA and their combinations with CHX and CTR are listed in ( Table 1). Twenty percent LA eradicated all enterococci biofilms after 30 seconds contact time. At 1 minute of exposure it eradicated them at a lesser concentration, 15%.

**Table 1:**
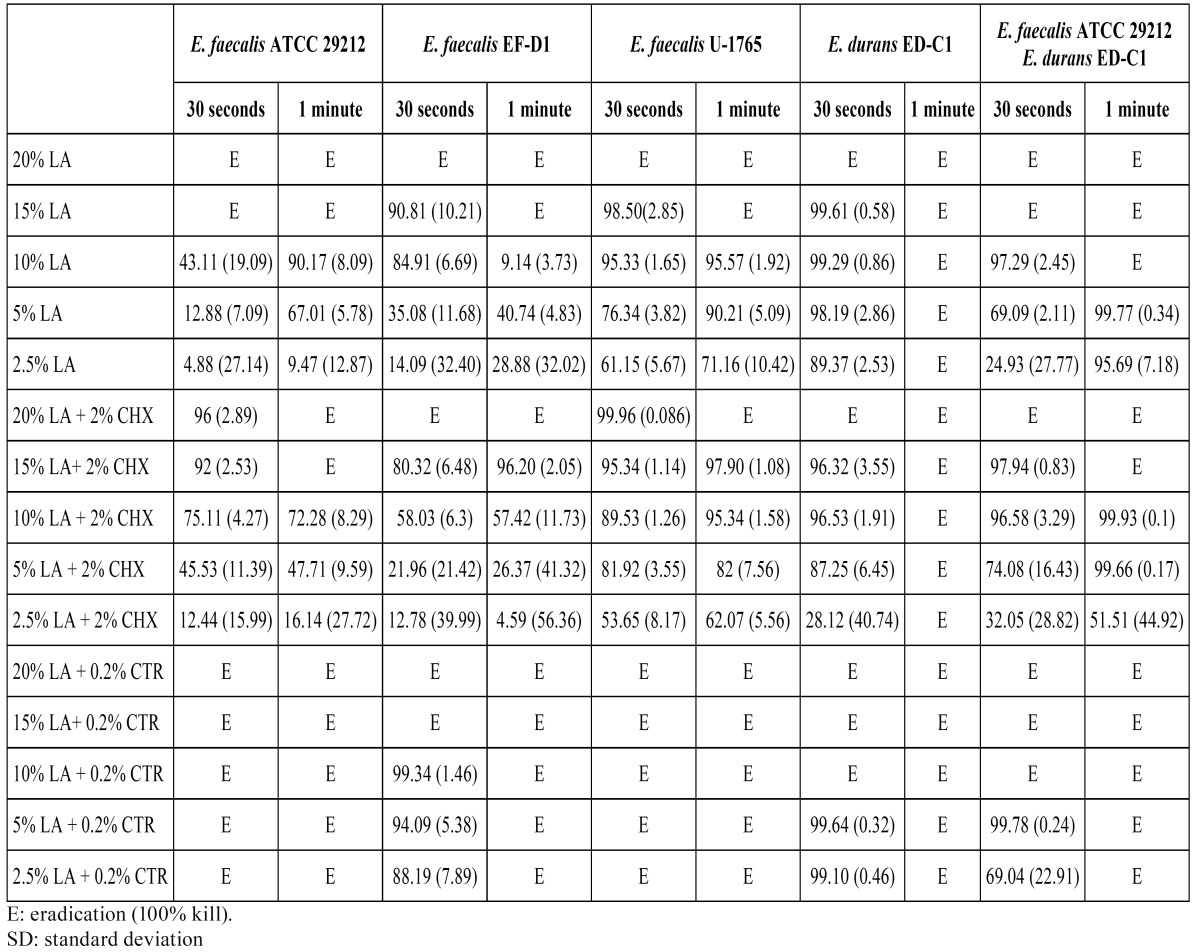
Kill percentage of enterococci biofilms by lactic acid (LA) and the combinations of 2% chlorhexidine (CHX) and 0.2% cetrimide (CTR) after 30 seconds and 1 minute contact time. Mean (SD).

Globally, the association of LA + 0.2% CTR achieved better results than LA alone, in contrast with the results obtained using LA + 2% CHX. The combinations of all the concentrations of LA + 0.2% CTR eradicated the biofilms of E. faecalis ATCC 29212 and U-1765 at 1 minute and 30 seconds of contact time. The biofilms of the other two strains and the dual-species biofilm experimented 100% kill, but only at 1 minute.

E. durans was very sensitive to exposure times of 1 minute, and was eradicated by all the tested solutions (alone and combined). The dual-species biofilm E. faecalis ATCC 29212 with E. durans gave intermediate values of the pure cultures.

## Discussion

The use of chelating agents associated with anti microbial and surfactant agents has been tested in attempts to remove the smear layer and control microorganisms from the root canal system. The lack of studies that evaluate enterococci biofilm susceptibility to LA chelating solution applied alone, or else in association with antimicrobials and surfactants, led us to explore the biofilm eradication capacity of different concentrations of LA and their combination with 2% CHX and 0.2% CTR.

The method selected was the MBEC™ HTP, as it has been previously used for the in vitro testing of the anti microbial activity of irrigants, allowing us to compare the results. In this device, four single and one dual enterococci strain biofilms were grown for 24 hours, all strains except one being E. faecalis, which are often isolated from necrotic or improperly filled root canal systems (17,18). Namely, the strains studied were E. faecalis ATCC 29212, a strain of reference widely used in anti microbial susceptibility studies (19); E. faecalis U-1765 isolated from a nosocomial infection; and two enterococci strains isolated from failed endodontic treatments, E. faecalis EF-D1 and E. durans ED-C1, the latter reportedly present in root canals (20). In addition, a dual-species biofilm was assayed (E. faecalis ATCC 29212 + E. durans ED-C1) due to the fact that endodontic infections are typically poly microbial (21).

LA was selected as it has been previously reported to effectively remove the smear layer from root canal (10) and to enhance the bond strength of Epiphany adhesive sealer to human dentin at 10 and 20% (11). In this study, the biofilms were eliminated by 20% LA after both contact times, and by a concentration of 15% after 1 minute. The anti microbial activity of LA may stem from its chemical nature as an organic acid. Organic acids decrease the internal pH of microbial cell through the ionization of dissociated acid molecules, and the disruption of substrate transport by altering cell membrane permeability (14). The low molecular weight of LA (90.08 Dalton) lead to smaller non-dissociated molecules that could enter the bacterial cells easily, and change the internal pH of the organisms.

LA was combined with 0.2% CTR, a cationic surfactant that reduces the surface tension of liquids (22). It has shown ability to eradicate E. faecalis biofilm in vitro (23) and, in human dentin, its anti microbial residual activity persisting over time (24). In this study, all combinations eradicated the enterococci biofilms after 1 minute and the biofilms of E. faecalis ATCC 29212 and E. faecalis U-1765 after 30 seconds. These findings indicate that this association enhances both solutions´ individual capacity to eradicate enterococci biofilm- 0.2% CTR has been previously shown to eradicate E. faecalis ATCC 29212 biofilm after 30 seconds and using the same methodology (25). Previous studies also show that 0.2% CTR associated with other organic acids at clinical concentrations proved able to eradicate biofilms of E. faecalis ATCC 29212 after 1 minute (associated with citric acid) and even after just 30 seconds (associated with maleic acid) (9). Our results confirm that this association is indeed effective.

The association of LA with CHX exhibited less antibiofilm efficacy than LA alone. This reduced effect could be due to the fact that CHX at 2% is not capable in itself of eradicating biofilms of E. faecalis either at 30 seconds or at 1 minute (23). The results of the association could be considered intermediate, that is, between the results obtained when the two solutions are applied separately. From the chemical standpoint, LA, with its anionic nature, could have been neutralized by the bicationic molecule of CHX. However, it was considered interesting a priori to evaluate this combination because CHX has high substantivity, and its association with another anionic molecule, fluoride, has been seen to reduce cariogenic microorganisms in the long term (25) without altering the efficacy of the fluorides in the control of dental caries (26).

The different results obtained between the various single biofilms and the dual-species biofilm lead us to weigh the importance of testing irrigating solutions not only against E. faecalis ATCC 29212 but also against wild strains and above all against the poly microbial biofilms that can be found in infected root canals. In this in vitro study, we were able to demonstrate that LA, aside from eliminating the smear layer (10) and improving the bond strength of Epiphany adhesive sealer (11) is capable of eradicating enterococci biofilm at a concentration of 20%. The combination of lower concentrations of this acid with 0.2% CTR achieved eradication after 1 minute of exposure. Ex vivo studies are needed to verify these results in the complexity of the root canal system.
